# Association of Intratumoral Microbiota with Prognosis in Patients with Lacrimal Gland Tumor

**DOI:** 10.3390/biomedicines13040960

**Published:** 2025-04-14

**Authors:** Jianping Hu, Yidi Yang, Yiyi Feng, Yu Yu, Xin Song, Renbing Jia

**Affiliations:** 1 Department of Ophthalmology, Shanghai Ninth People’s Hospital, Shanghai Jiao Tong University School of Medicine, Shanghai 200025, China; jphu1993@hotmail.com (J.H.); yidi321@163.com (Y.Y.); yiyifeng0216@outlook.com (Y.F.); shsmu_yuyu@163.com (Y.Y.); 2 Shanghai Key Laboratory of Orbital Diseases and Ocular Oncology, Shanghai 200025, China

**Keywords:** intratumoral microbiota, malignant lacrimal gland tumor, prognosis

## Abstract

**Background**: While intratumoral microbiota have been identified in various cancers, their presence and clinical significance in lacrimal gland tumors remain largely unexplored. This study investigates the existence, composition, and potential clinical significance of intratumoral bacteria in lacrimal gland tumors. **Methods**: High-throughput 16S rDNA sequencing was performed on tumor DNA extracted from 89 paraffin-embedded tissues from patients with lacrimal gland tumors. Diversity analysis and LEfSe differential analysis were conducted to identify tumor-type-specific bacterial taxa. LASSO regression and the Cox proportional hazards models were used to analyze the relationship between intratumoral microbiota and prognosis. **Results**: Significant differences in the β diversity of intratumoral microbiota were observed across adenoid cystic carcinoma (ACC), carcinoma ex pleomorphic adenoma (CXPA), pleomorphic adenoma (PA), and IgG4-related disease (IgG4-RD) patients. After FDR correction, *Garicola*, *Prevotella*, *Polaribacter*, and *Helicobacter* were notably enriched in the tumors of ACC, CXPA, PA, and IgG4-RD patients, respectively. Importantly, patients with malignant lacrimal gland tumors who experienced relapse, distant metastasis, or death had significantly higher α diversity within their tumors. Furthermore, specific genera, such as *Roseburia* and *Alloprevotella*, were particularly associated with poorer prognosis in patients with malignant lacrimal gland tumors. **Conclusions**: This study provides a comprehensive analysis of microbial profiles in lacrimal gland tumors, highlighting distinct microbial characteristics across tumor types. Our findings suggest that intratumoral bacterial diversity and specific genera may serve as potential prognostic markers for malignant lacrimal gland tumors.

## 1. Introduction

Lacrimal gland tumors account for approximately 6–12% of orbital lesions, with 20–28% classified as primary epithelial tumors, while the remainder consist of inflammatory and lymphatic lesions [1,2]. Among epithelial tumors, 50–55% are benign, while 45–50% are malignant [1,2]. The most common benign tumors are pleomorphic adenomas (PA), which have significant potential for local recurrence and malignant transformation into carcinoma ex pleomorphic adenoma (CXPA). Adenoid cystic carcinoma (ACC) is the most prevalent malignant tumor, accounting for 40–60% of cases, followed by CXPA at 18–20% [1,2]. Despite the rarity of lacrimal gland tumors, they often affect relatively young individuals and can have a severe prognosis [1]. However, there remains a lack of reliable markers to predict the progression and prognosis of these tumors.

Lacrimal gland tumors typically present with symptoms related to the mass effect of the growing tumor, including globe displacement (usually medially and downward), decreased ocular motility, diplopia, ptosis, and a palpable mass [3,4]. ACC is an aggressive malignancy characterized by perineural invasion, making pain a hallmark symptom that distinguishes it from other lacrimal gland tumors [5]. Symptoms of ACC progress rapidly, typically within six months, with significant ocular displacement and functional impairment [6]. CXPA develops from a pre-existing PA and is often identified when a previously slow-growing, painless mass undergoes sudden rapid enlargement. Its clinical presentation reflects malignant transformation, with increased aggressiveness and a shorter symptom duration before diagnosis [6,7]. PA, the most common benign lacrimal gland tumor, usually progresses over one to two years before patients seek medical attention. It manifests as a painless, gradually enlarging mass that displaces the globe but has minimal impact on ocular motility [6,7]. In contrast, IgG4-related disease (IgG4-RD) of the lacrimal gland presents as chronic, painless swelling, often with associated ptosis [8]. Unlike neoplastic lesions, IgG4-RD frequently affects both eyes and may involve systemic manifestations, mimicking inflammatory or autoimmune disorders rather than true tumors [8].

Microbiota, including bacteria, fungi, viruses, and other eukaryotes, play pivotal roles in human health, including in cancer development [9,10]. Previous studies have highlighted the involvement of gut microbiota in cancer pathogenesis. Recently, intratumoral microbiota have also been identified in various cancer types, such as pancreatic, lung, and breast cancers [11]. These intratumoral microbiota have shown a strong correlation with cancer progression and prognosis [12]. Notably, infectious microbiota are linked to over 16% of cancer incidence worldwide [13], suggesting the involvement of several microorganisms in the pathogenesis of ocular adnexal malignancies [14]. Therefore, exploring the role of microbiota in lacrimal gland tumors is crucial for a comprehensive understanding of their pathogenesis and potential prognostic markers.

In this study, 16S rDNA sequencing was employed to investigate the presence of intratumoral bacteria and their clinical significance in patients with lacrimal gland lesions, including malignant tumors, such as ACC and CXPA, benign tumors, such as PA, and inflammatory lesions in IgG4-RD.

## 2. Materials and Methods

### 2.1. Study Population

In this study, a total of 89 paraffin-embedded tissue samples were obtained from Shanghai Ninth People’s Hospital (Shanghai, China). The samples included 23 tissues from ACC patients (12 male, 11 female), 21 tissues from PA patients (11 male, 10 female), and 21 tissues from CXPA patients (13 male, 8 female), all collected during surgical resection prior to any radiotherapy or chemotherapy. Additionally, 24 tissue samples from IgG4-RD patients (17 male, 7 female) were obtained through diagnostic biopsies before any treatment. All ACC and CXPA patients were restaged based on the 8th American Joint Committee on Cancer staging system, with any discrepancies resolved through consensus. Ethical approval for the use of tissue specimens in this study was obtained from Shanghai Ninth People’s Hospital, and written informed consent was acquired from each participating patient.

### 2.2. DNA Extraction

DNA was isolated using the QIAamp DNA FFPE Tissue Kit (QIAGEN, Hilden, Germany) following the manufacturer’s instructions. Briefly, the tissues were dissolved in xylene to remove paraffin. The sample was then lysed under denaturing conditions with a short proteinase K digestion. After incubation at 90 °C, DNA was bound to the membrane, allowing contaminants to flow through. Finally, DNA was eluted in water and immediately stored at −20 °C. To minimize contamination, nonenzymatic kit components, pipette tips, and sterile pipettes were UV-irradiated for over an hour prior to use.

### 2.3. 16S rDNA Sequencing

PCR amplification of the hypervariable regions 3–4 (V3–V4) of the bacterial 16S rRNA gene was performed using universal primers (343F: 5′-TACGGRAGGCAGCAG-3′; 798R: 5′-AGGGTATCTAATCCT-3′). The PCR products were purified using Agencourt AMPure XP beads (Beckman Coulter Co., Brea, CA, USA) and quantified with the Qubit dsDNA assay kit. Sequencing was then performed on an IlluminaNovaSeq6000 with two paired-end read cycles of 250 bases each (Illumina Inc., San Diego, CA, USA).

### 2.4. Contamination Removal Procedure

To ensure the reliability of our analysis, we applied the RIDE checklist, a comprehensive framework for addressing contamination in low-biomass samples [15]. Initially, ASVs identified as mitochondrial or chloroplast sequences from the host were removed using KneadData. To minimize false positives, we accounted for three different batch effects: sequencing library batch, DNA extraction batch, and PCR amplification batch. Further refinement included removing singleton ASVs and retaining only ASVs detected in at least two patients (Figure S1). Additionally, a nonparametric, exact binomial test was applied to assess the prevalence of each taxon, using a null probability of 0.01 and comparing taxon prevalence in samples with their prevalence in the corresponding negative controls. Only taxa with a *p*-value below 0.05 across all batch comparisons were included in the subsequent analyses.

### 2.5. Bioinformatic Analysis

The paired-end reads were preprocessed using Cutadapt software (version 4.0) to detect and remove adapters. Low-quality sequences were denoised, merged, and chimera reads were detected and removed using DADA2 with default parameters in the QIIME 2 package [16]. The output representative read of each ASV was selected using QIIME 2. A contamination removal procedure was established to filter contaminant ASV (Figure S1). Microbial diversity was assessed using alpha diversity metrics, including the Chao1 index, Simpson index, and Shannon index [17]. The UniFrac distance matrix was calculated using QIIME software (version 1.8.0) and used for unweighted and weighted UniFrac principal coordinates analysis (PCoA) [18].

For differential abundance analysis, LEfSe analysis was employed to identify taxa that showed significant differences between groups [19]. LEfSe analysis was performed using the LEfSe tool (http://huttenhower.sph.harvard.edu/lefse/, accessed on 30 August 2023), with a logarithmic LDA score threshold of >2.0 and a *p*-value cutoff of <0.05. This approach facilitated the identification of key microbial features associated with different tumor types and their potential as prognostic biomarkers.

### 2.6. Statistics Analysis

The results are presented as mean ± standard deviation (SD). Differences in baseline characteristics among study participants with different lacrimal gland tumors were evaluated using the χ^2^ test for categorical variables and analysis of variance (ANOVA) for continuous variables. Multiple group comparisons were conducted using one-way ANOVA and the Kruskal–Wallis test, followed by Dunn’s correction. LASSO regression was applied to select bacterial genera associated with prognosis by penalizing less relevant features and minimizing overfitting. Cox proportional hazards regression models were used to estimate HRs and 95%CIs to assess the association between intratumoral microbiota and the risk of poor prognosis. For analysis of two independent groups, unpaired *t*-tests and Mann–Whitney U tests were employed. To control for multiple comparisons, the FDR correction was applied. Statistical analyses were carried out using GraphPad Prism 7 software (GraphPad Software, Inc., San Diego, CA, USA) and R software (version 4.3.0). Significance was determined at *p* < 0.05.

## 3. Results

### 3.1. Diversity of Intratumoral Microbiota in Patients with Lacrimal Gland Tumors

**Figure 1:**
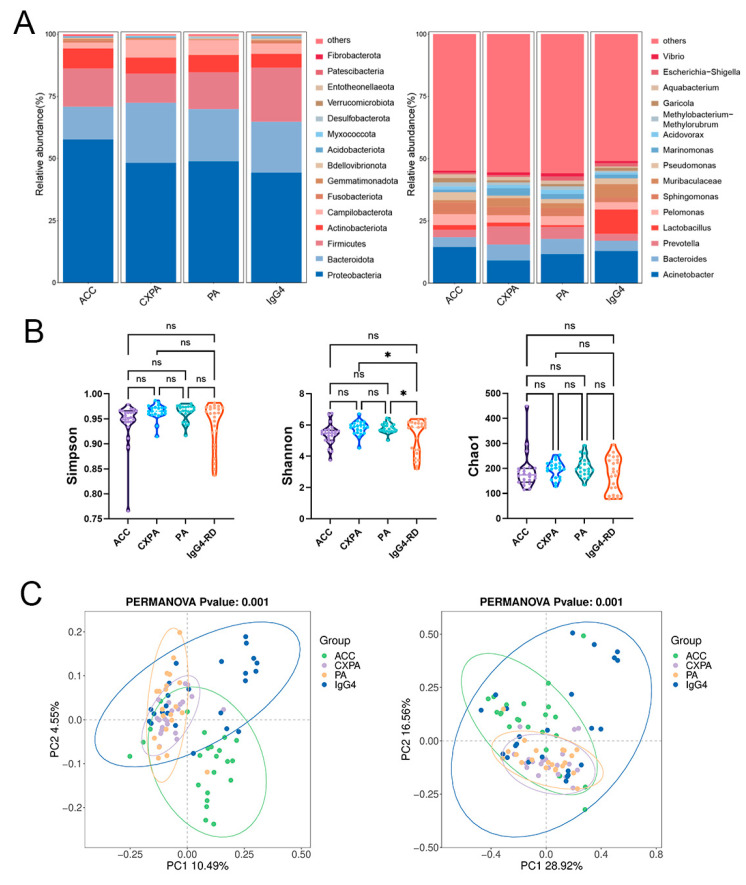
Altered microbiota composition in the tissues of patients with ACC (*n* = 23), CXPA (*n* = 21), PA (*n* = 21), and IgG4-RD (*n* = 24). (**A**) Phylum- and genus-level taxonomic distributions of intratumoral microbiota in patients with ACC, CXPA, PA, or IgG4-RD. (**B**) Alpha diversity analysis of intratumoral microbiota in patients with ACC, CXPA, PA, or IgG4-RD. (**C**) Principal coordinate analysis (PCoA) using unweighted (left) and weighted (right) UniFrac metrics of samples based on the relative abundance of OTUs. Data are presented as mean ± SD. ns: *p* > 0.05, * *p* < 0.05.

Our primary objective was to identify potential variations in microbial composition across different types of lacrimal gland lesions. Paraffin-embedded tissue samples were obtained from 23 ACC patients, 21 PA patients, 21 CXPA patients, and 24 IgG4-RD patients, with blank paraffin sections serving as the negative control. The demographic and clinical characteristics of the patients are detailed in Supplementary Table S1. Significant differences in microbial composition were observed among the four groups at both the phylum and genus levels (Figure 1A). Although the Simpson and Shannon indices were higher in CXPA and PA compared to IgG4-RD, no other significant differences in alpha diversity were noted among the ACC, CXPA, PA, and IgG4-RD groups (Figure 1B). Principal coordinate analysis (PCoA) revealed distinct microbial profiles for ACC, PA, and the inflammatory IgG4-RD, while CXPA exhibited a microbial composition similar to PA (Figure 1C). This similarity is likely due to CXPA arising from the malignant transformation of PA.

### 3.2. Intratumoral Microbiota Differences in Patients with Lacrimal Gland Tumors

**Figure 2:**
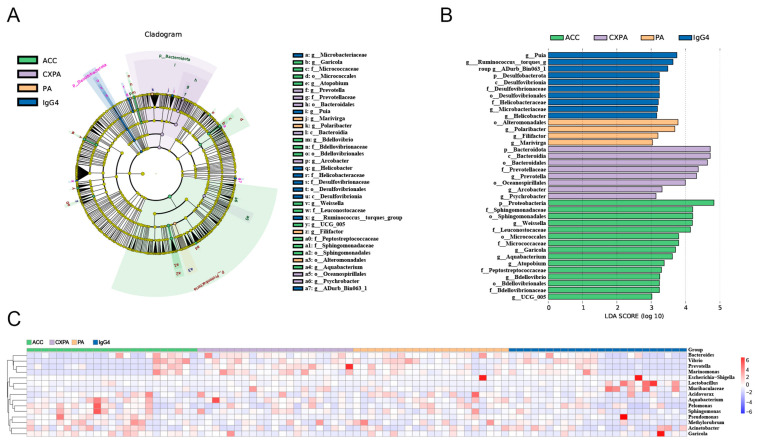
Altered microbiota composition in the tissues of patients with ACC (*n* = 23), CXPA (*n* = 21), PA (*n* = 21), and IgG4-RD (*n* = 24). (**A**) Differentially abundant taxonomic clades with an LDA score > 2.0 and a *p*-value < 0.05. (**B**) Linear discriminant analysis (LDA) effect size (LEfSe) analysis in patients. (**C**) Heatmap of the top 15 differentially enriched genera in patients. ACC (blue), CXPA (orange), PA (purple), or IgG4-RD (green).

To explore differences in bacterial taxa among patients with various lacrimal gland lesions, we conducted a LEfSe (linear discriminant analysis effect size) analysis. The analysis identified 14 bacterial taxa enriched in ACC tissues, 8 in CXPA tissues, 4 in PA tissues, and 10 in IgG4-RD tissues (Figure 2B). Cladograms across six taxonomic levels (from kingdom to genus) were generated, illustrating these differences. In ACC tissues, notable taxa included the orders *Sphingomonadales*, *Bdellovibrionales*, and *Micrococcales*, with significant contributions from families such as *Sphingomonadaceae*, *Peptostreptococcaceae*, *Leuconostocaceae*, and *Micrococcaceae*. In CXPA tissues, the class *Bacteroidia*, along with the orders *Oceanospirillales*, *Bacteroidales*, and the family *Prevotellaceae*, showed large effect sizes. In PA tissues, the order *Alteromonadales* was prominent, while in IgG4-RD tissues, the class *Desulfovibrionia*, order *Desulfovibrionales*, and families *Desulfovibrionaceae* and *Helicobacteraceae* had significant effect sizes (Figure 2A).

Additionally, the Kruskal–Wallis test and log LDA scores were used to compare the relative abundance of microbiota and identify significantly different bacterial taxa across various lacrimal gland tumors. At the genus level, six genera were enriched in ACC tissues, three genera in CXPA tissues, two in PA tissues, and four genera were enriched in IgG4-RD tissues. The top 15 differentially enriched genera in tumor tissues from patients are shown in Figure 2C. After applying FDR correction, only *Garicola*, *Prevotella*, *Polaribacter*, and *Helicobacter* remained significantly enriched in ACC, CXPA, PA, and IgG4-RD tissues, respectively (Table S2).

### 3.3. Intratumoral Microbiota Is Associated with the Poor Prognosis of Malignant Lacrimal Gland Tumors

**Figure 3:**
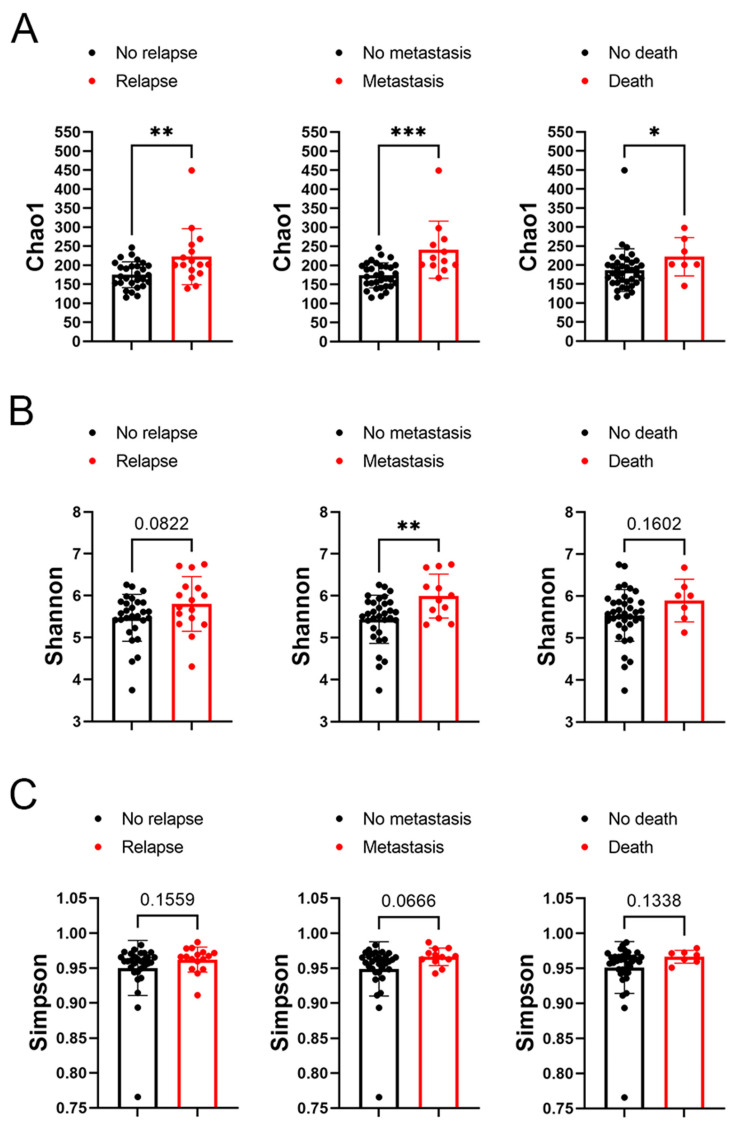
Association between alpha diversity of intratumoral microbiota and prognosis in patients with malignant lacrimal tumors (N = 44). (**A**) The Chao1 index of intratumoral bacteria in patients with or without tumor relapse, metastasis, or death. (**B**) The Shannon index of intratumoral bacteria in patients with or without tumor relapse, metastasis, or death. (**C**) The Simpson index of intratumoral bacteria in patients with or without tumor relapse, metastasis, or death. Data are shown as mean ± SD. * *p* < 0.05, ** *p* < 0.01, *** *p* < 0.001.

**Figure 4:**
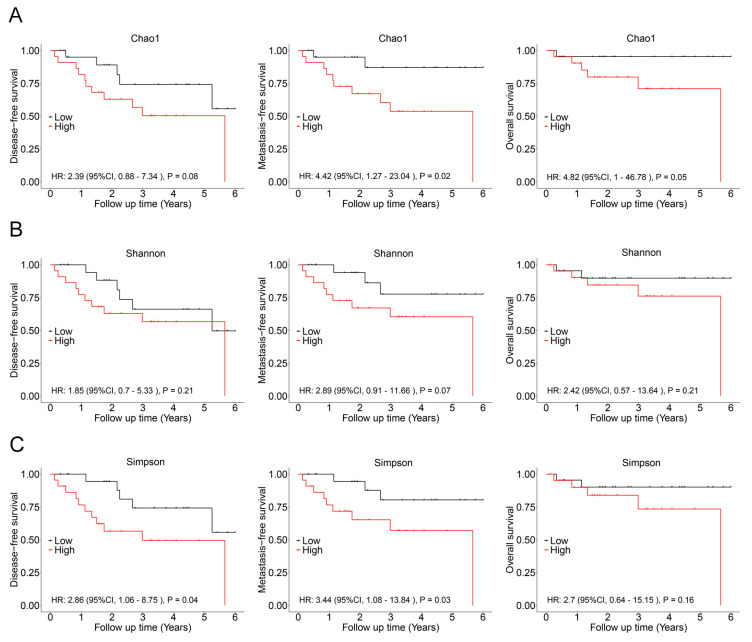
Association between intratumoral bacterial alpha diversity and prognosis in patients with malignant lacrimal tumors (N = 44). (**A**) Kaplan–Meier curves depicting disease-free survival, metastasis-free survival, and overall survival in patients with a high Chao1 index (>189.90). (**B**) Kaplan–Meier curves depicting disease-free survival, metastasis-free survival, and overall survival in patients with a high Shannon index (>0.962). (**C**) Kaplan–Meier curves depicting disease-free survival, metastasis-free survival, and overall survival in patients with a high Simpson index (>5.614). *p*-values were analyzed using the log-rank test, and HRs and 95%CIs were calculated using Cox regression analysis, adjusted for age, sex, tumor stage, and therapy.

In subsequent analysis, the correlation between the alpha diversity of intratumoral microbiota and the prognosis of malignant lacrimal gland tumors was explored. With a median follow-up period of 2.27 years, 16 out of 44 patients (including those with CXPA and ACC) experienced relapse, 12 developed metastasis, and 7 succumbed to the disease. The results indicated that the Chao1 index of intratumoral microbiota was significantly higher in patients who experienced relapse, metastasis, or death (Figure 3). To further investigate this association, Chao1, Shannon, and Simpson indices above the median were defined as high microbial diversity, respectively. A high Chao1 index in tumor tissue was associated with an increased risk of metastasis (HR: 4.42, 95%CI: 1.27–23.04, *p* = 0.02) and mortality (HR: 4.82, 95%CI: 1.00–46.78, *p* = 0.05; Figure 4A). Additionally, a higher Chao1 index was associated with a trend toward an increased risk of relapse (HR: 2.39, 95%CI: 0.88–7.34, *p* = 0.08; Figure 4A). Moreover, a high Simpson index in tumor tissue was also linked to a greater risk of relapse (HR: 2.86, 95%CI: 1.06–8.75, *p* = 0.04) and metastasis (HR: 3.44, 95%CI: 1.08–13.84, *p* = 0.03; Figure 4C). The Shannon index was not significantly associated with recurrence, metastasis, or mortality (Figure 4B). Similar trends were observed when analyzing ACC and CXPA patients separately (Figures S2 and S3). These findings suggest that high microbial diversity may serve as a potential prognostic marker for malignant lacrimal gland tumors.

Furthermore, 6 genera were found to be increased in patients with relapse (Figure S4A and Table S3), 11 genera were elevated in patients with metastasis (Figure S4B and Table S4), and 12 genera were enriched in patients who succumbed to the disease (Figure S4C and Table S5). Notably, the genera *Roseburia* and *Marinifilum* were increased in patients experiencing either relapse, metastasis, or death (Figure S4D). The genera *Brachybacterium* and *Butyricimonas* were elevated in patients with either relapse or metastasis, while the genera *Cobetia* and *Lachnospiraceae_UCG-010* were enriched in patients with either metastasis or death (Figure S4D). However, after applying FDR correction, no genera remained significantly enriched in patients with poor prognosis (Figure S4D).

**Figure 5:**
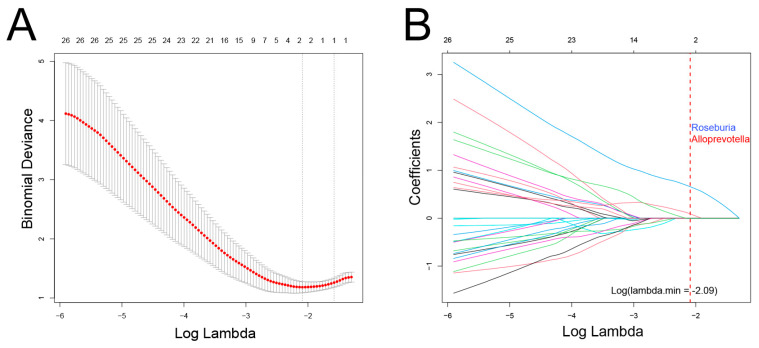
Association between intratumoral microbiota and prognosis in patients with malignant lacrimal tumors (N = 44). (**A**) Cross-validation results of the LASSO regression analysis. (**B**) LASSO coefficient path. Two genera were selected when log(λ) = −2.09.

**Figure 6:**
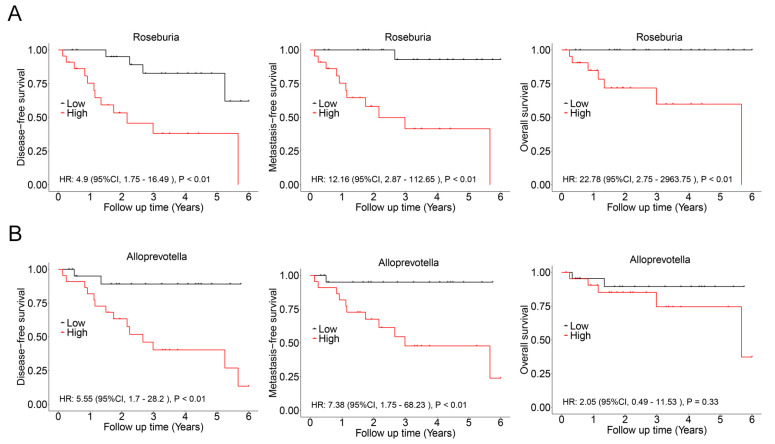
Association between intratumoral microbiota and prognosis in patients with malignant lacrimal tumors (N = 44). (**A**) Kaplan–Meier curves depicting disease-free survival, metastasis-free survival, and overall survival in patients with a high relative abundance of *Roseburia*. (**B**) Kaplan–Meier curves depicting disease-free survival, metastasis-free survival, and overall survival in patients with a high relative abundance of *Alloprevotella*. *p*-values were analyzed using the log-rank test, and HRs and 95%CIs were calculated using Cox regression analysis, adjusted for age, sex, tumor stage, and therapy.

In this cohort, there was a significant overlap among patients who experienced recurrence, metastasis, and death. Consequently, these events were combined, resulting in a total of 17 cases classified as having a poor prognosis. LASSO regression analysis was then conducted to identify bacterial genera associated with poor prognosis, revealing *Roseburia* and *Alloprevotella* as potential contributors to recurrence, metastasis, or mortality (Figure 5). These genera were further validated using Cox regression, which revealed that *Roseburia* was significantly associated with recurrence (HR: 4.9, 95%CI: 1.75–16.49, *p* < 0.01), metastasis (HR: 12.16, 95%CI: 2.87–112.65, *p* < 0.01), and mortality (HR: 22.78, 95%CI: 2.75–2963.75, *p* < 0.01). Similarly, *Alloprevotella* was associated with recurrence (HR: 5.55, 95%CI: 1.7–28.2, *p* < 0.01) and metastasis (HR: 7.38, 95%CI: 1.75–68.23, *p* < 0.01; Figure 6). Consistent findings were observed when analyzing ACC and CXPA patients separately, further reinforcing the potential prognostic value of these genera (Figures S5 and S6).

### 3.4. Intratumoral Microbiota Is Associated with the Recurrence and Malignancy of PA After Surgery

In salivary glands, CXPA occurs in approximately 6.2% of patients with PA. Currently, there are no available biomarkers to predict the risk of malignant transformation of benign PA. This study aimed to investigate whether the composition of intratumoral microbiota is linked to the malignant transformation of benign PA. Unfortunately, in this PA cohort, no patients developed CXPA, and no significant differences were found in microbiota diversity between CXPA and PA tissues (Figure 1B). However, among the CXPA patients in our cohort, eight had a prior PA diagnosis and had undergone surgery. In these patients, six genera were found to be enriched, particularly *Butyricimonas* and *Cobetia*, which have been associated with the poor prognosis in ACC and CXPA patients (Table S6). However, no differences in alpha diversity were observed in these surgically treated patients (Figure S7).

## 4. Discussion

This study provides a comprehensive analysis of the microbial landscape in various lacrimal gland lesions, including both malignant and benign types. Through 16S rDNA sequencing, we identified significant differences in the microbial diversity between lacrimal gland tumors, such as ACC, CXPA, PA, and IgG4-RD. Our findings highlight the potential role of intratumoral microbiota in the malignant progression of lacrimal gland tumors.

Numerous studies have highlighted the presence of intratumoral microbiota in various cancer types, including bacteria, viruses, fungi, and mycoplasmas, while implementing strict contamination control strategies to ensure the validity of findings [20]. These microbial communities have been shown to play significant roles in tumor progression, immune modulation, and even therapy outcomes. In this study, we found that *Garicola* and *Prevotella* were enriched in tumor tissues from ACC and CXPA patients, respectively. *Prevotella* has previously been observed to increase in tumor tissues from patients with gastric cancer [21], lung cancer [22], bladder cancer [23], and cervical cancer [24]. Moreover, *Prevotella* was found to be depleted in breast cancer tissues after patients underwent neoadjuvant chemotherapy [25]. These findings suggest a potential correlation between *Prevotella* and the progression of various malignant cancers. Furthermore, our results are the first to show an increase in *Garicola* in tumor tissues from ACC patients compared to other lacrimal gland lesions. *Garicola* is a genus belonging to the family *Micrococcaceae,* which was observed enriched in endometrial cancer compared to benign uterine lesions [26].

Intratumoral bacteria have been linked to clinical outcomes and prognosis across various cancers. For instance, Qiao et al. confirmed that a higher intratumoral bacterial load in nasopharyngeal carcinoma (NPC) tissues was correlated with poor survival, as it was negatively associated with T-lymphocyte infiltration [12]. Similarly, in pancreatic ductal adenocarcinoma (PDAC), a greater abundance of *Sphingomonas* and *Megasphaera* was positively linked to better prognosis, while a higher abundance of *Clostridium* was associated with poorer outcomes [27]. In hepatocellular carcinoma (HCC), the presence of *Firmicutes* and *Actinobacteria* was associated with increased tumor volume, cirrhosis, and worse prognosis [28]. Furthermore, elevated levels of *Fusobacterium nucleatum* in esophageal squamous cell carcinoma (ESCC) have been connected to poorer recurrence-free survival [29]. Increased proportions of *Roseburia* and *Blautia* have been observed in patients with lung cancer recurrence, reinforcing the link between specific bacteria and cancer outcomes [30]. In laryngeal squamous cell carcinoma, a high relative abundance of *Alloprevotella* was significantly linked to a higher risk of recurrence [31].

This study demonstrated that high microbial diversity within tumor tissues is significantly associated with poor prognosis in malignant lacrimal gland tumors. This association was reflected in patients with higher Chao1 and Simpson indices, showing increased risks of relapse, metastasis, and mortality. Furthermore, we confirmed that specific genera, such as *Roseburia* and *Alloprevotella*, were strongly linked to poor outcomes, further supporting the role of intratumoral microbiota as a prognostic marker. Interestingly, the genera *Clostridium*, *Fusobacterium nucleatum*, *Roseburia*, *Blautia*, and *Alloprevotella*, which were associated with poor prognosis, are all anaerobic bacteria. This observation aligns with the widely recognized concept that tumor hypoxia is linked to poor clinical outcomes [32]. Hypoxic conditions in the tumor microenvironment promote immune evasion, angiogenesis, metabolic reprogramming, and resistance to therapy, ultimately leading to tumor progression and metastasis [32]. This may be precisely the reason why anaerobic bacteria are enriched in the tumor tissues of patients with poor prognosis.

Various studies have thoroughly investigated the role of the microbiome in tumorigenesis and cancer progression, uncovering various underlying mechanisms. One key mechanism involves DNA damage, which can lead to increased mutation rates and promote cancer development. For example, *Escherichia coli* can induce DNA double-strand breaks, disrupting the normal cell cycle and contributing to tumorigenesis [33]. Similarly, *Pseudomonas aeruginosa* can cause DNA damage and enhance the accumulation of reactive oxygen species through its toxin production, further complicating cancer pathology [34]. Additionally, *Porphyromonas gingivalis* has been linked to increased mutation rates in oncogenes, such as KRAS and TP53, which play crucial roles in the progression of PDAC [35,36]. Another crucial aspect is the modulation of tumor signaling pathways by microbial communities. *Fusobacterium nucleatum* has been reported to activate the TLR4-mediated autophagy pathway in colorectal cancer cells, which may contribute to drug resistance [37]. Furthermore, bacterial metabolites have been shown to interact with GPCRs, engaging in various downstream anti- or pro-cancer signaling pathways [38].

Beyond direct cellular effects, the microbiome stimulates immune cells and influences the recruitment and aggregation of adhesion molecules and cytokines, thereby affecting cancer cell proliferation [39]. Bacterial metabolites, enzymes, and toxins can exert their effects both locally and systemically, contributing to chronic inflammation. Additionally, the microbiome can directly shape the immune microenvironment of tumors [40,41,42]. *Fusobacterium nucleatum,* in particular, has been associated with the establishment of a tumor immunosuppressive microenvironment through the targeted recruitment of tumor-infiltrating myeloid cells, tumor-associated macrophages, and myeloid-derived suppressor cells [43,44]. Moreover, cytokines and immunosuppressive molecules serve as independent regulators of tumor immunity, with interventions targeting these pathways showing promise in impeding the progression of melanoma [45].

Microbial metabolites are crucial mediators of host–microbe interactions and play a significant role in shaping the immune environment and various physiological functions. These metabolites contribute to maintaining host mechanisms, including nutritional pathways and detoxification, and can significantly impact the efficacy of chemotherapy and tumor metastasis [46,47]. Notably, butyrate, produced by genera such as *Coprococcus*, *Roseburia*, and *Butyricimonas*, has been implicated in various aspects of tumor biology [48,49]. Previous studies have shown that butyrate can induce the proliferation of colonic epithelial cells with deficiencies in MLH1 or MSH2, enhancing β-catenin activity [48,49]. Furthermore, butyrate promotes liver cancer metastasis by increasing the expression of H19 in tumor cells through the inhibition of HDAC2, leading to increased H3K27 acetylation at the H19 promoter and inducing M2 macrophage polarization [30]. In this study, we observed a significant enrichment of butyrate-producing bacteria in patients with poor prognosis, emphasizing the potential impact of butyrate on tumor progression. The presence of these bacteria may contribute to unfavorable outcomes by modulating inflammation, regulating apoptosis, and influencing cell proliferation.

The presence of microbiota in tumors, including bacteria, fungi, mycoplasma, and viruses, has been extensively confirmed by numerous studies [20]. However, their origin remains unclear. Current research suggests several potential sources of intratumoral microbes. Firstly, they may originate from adjacent normal tissues, as tumors can compromise mucosal barrier integrity by altering anatomy, thereby enabling microbial invasion facilitated by local immunosuppression and inflammation [50]. Moreover, mucous membranes that directly communicate with the external environment (e.g., genitourinary system, digestive tract, lungs, and skin) harbor abundant microbiota [51]. Additionally, the presence of microbes in tumors may also colonize through blood circulation [52]. In the case of the lacrimal gland, it communicates directly with the ocular surface through the lacrimal duct, suggesting that the microorganisms in the tumor may primarily originate from the ocular surface. Unfortunately, we do not have matching patient ocular surface microbiome information to confirm this hypothesis.

## 5. Limitations

This study has several limitations that should be acknowledged. Firstly, technical constraints hindered the complete assembly of microbial genomes through metagenomic sequencing, particularly in tumors with low biomass. Despite rigorous quality control measures, paraffin-embedded samples remain susceptible to contamination from environmental microorganisms. Secondly, while we observed a potential negative correlation between high bacterial load and prognosis in ACC and CXPA patients, larger cohorts are needed for validation. Additionally, the substantial overlap between relapse and metastasis cases in this cohort limits the ability to distinguish their independent prognostic significance based on microbial signatures. External validation in independent cohorts is essential to confirm these findings. Further research is also needed to clarify the relationship between microbial load and patient outcomes, as well as the underlying biological mechanisms.

## 6. Conclusions

To our knowledge, this study represents a new comprehensive exploration of microbial profiles in lacrimal gland tumors. Our findings revealed that different types of lacrimal gland tumors harbor distinct microbial compositions, suggesting a potential role for the tumor microbiome in disease pathogenesis. Notably, we identified significant differences in both intratumoral bacterial diversity and taxonomic profiles between benign and malignant tumors. Furthermore, our results suggested a potential association between intratumoral microbial diversity and prognosis, with specific bacterial genera, such as *Roseburia* and *Alloprevotella*, being enriched in malignant tumors. These genera may serve as biomarkers for disease progression, highlighting the prognostic significance of the tumor microbiome. Overall, our study underscores the critical role of microbial dysbiosis in lacrimal gland malignancies and suggests that microbial signatures could provide novel insights into tumor biology, risk stratification, and potential therapeutic interventions. Further studies are warranted to elucidate the mechanistic interplay between the microbiome and lacrimal gland tumor progression.

## Data Availability

Related data can be acquired from the corresponding authors upon reasonable request.

## Supplementary Materials

The following supporting information can be downloaded at: https://www.mdpi.com/article/10.3390/biomedicines13040960/s1. Figure S1. The rarefaction curve generated based on 16S rRNA sequencing data after the contamination removal procedure. Figure S2. Association of intratumoral microbiota with the prognosis in patients with ACC (N = 23). Figure S3. Association of intratumoral microbiota with the prognosis in patients with CXPA (N = 21). Figure S4. Association of intratumoral microbiota with prognosis in patients with malignant lacrimal tumors (N = 44). Figure S5. Association of intratumoral microbiota with the prognosis in patients with ACC (N = 23). Figure S6. Association of intratumoral microbiota with the prognosis in patients with CXPA (N = 21). Figure S7. Association of intratumoral microbiota with recurrence and malignancy in PA patients following surgery. Table S1. The clinical characteristics of patients with lacrimal gland tumors. Table S2. List of significantly different microbiota between ACC, CXPA, PA and IgG4-RD patients. Table S3. The relative abundance of significantly different microbiota between patients with or without relapse. Table S4. The relative abundance of significantly different microbiota between patients with or without metastasis. Table S5. The relative abundance of significantly different microbiota between patients with or without death. Table S6. The relative abundance of significantly different microbiota between CXPA patients with or without a history of surgery.
