# Supplementary material for: Association of Intratumoral Microbiota with Prognosis in Patients with Lacrimal Gland Tumor
Source: Biomedicines. 2025 Apr 14;13(4):960. doi: 10.3390/biomedicines13040960 (PMC12024857; doi:10.3390/biomedicines13040960)
Supplement: Supplementary file 1 [file biomedicines-13-00960-s001.zip › biomedicines-3527749-supplementary.pdf]

Supplementary figures and tables

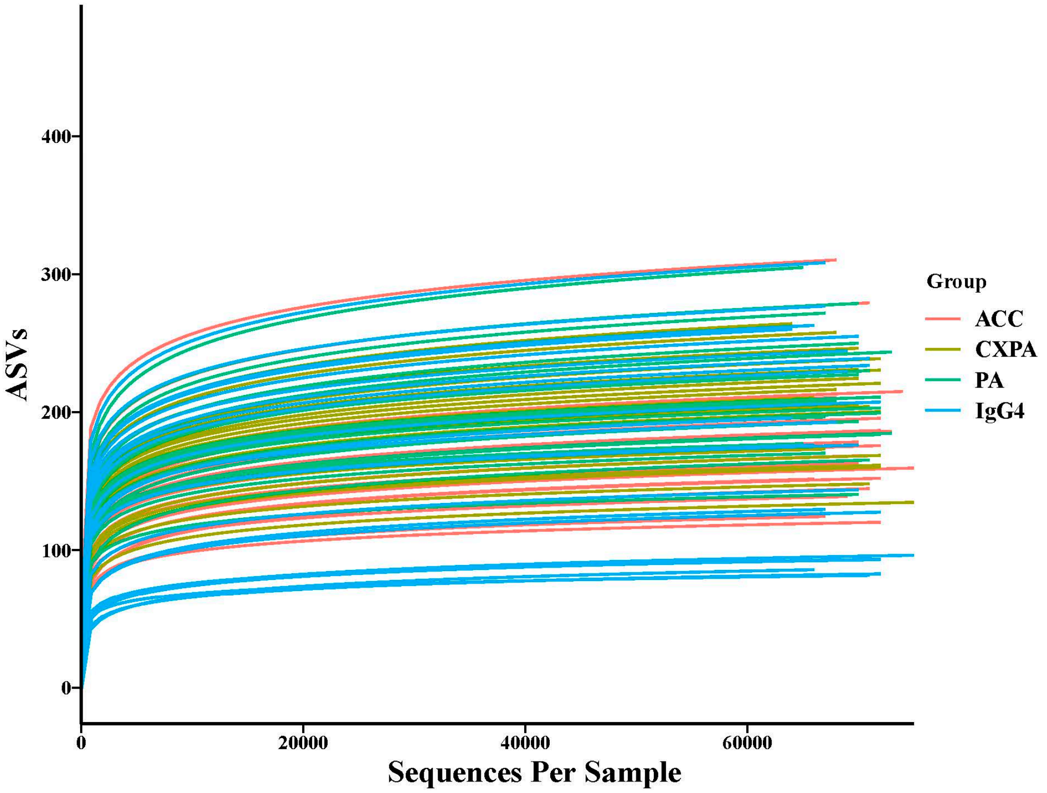

Figure S1. The rarefaction curve generated based on 16S rRNA sequencing data after the contamination removal procedure.

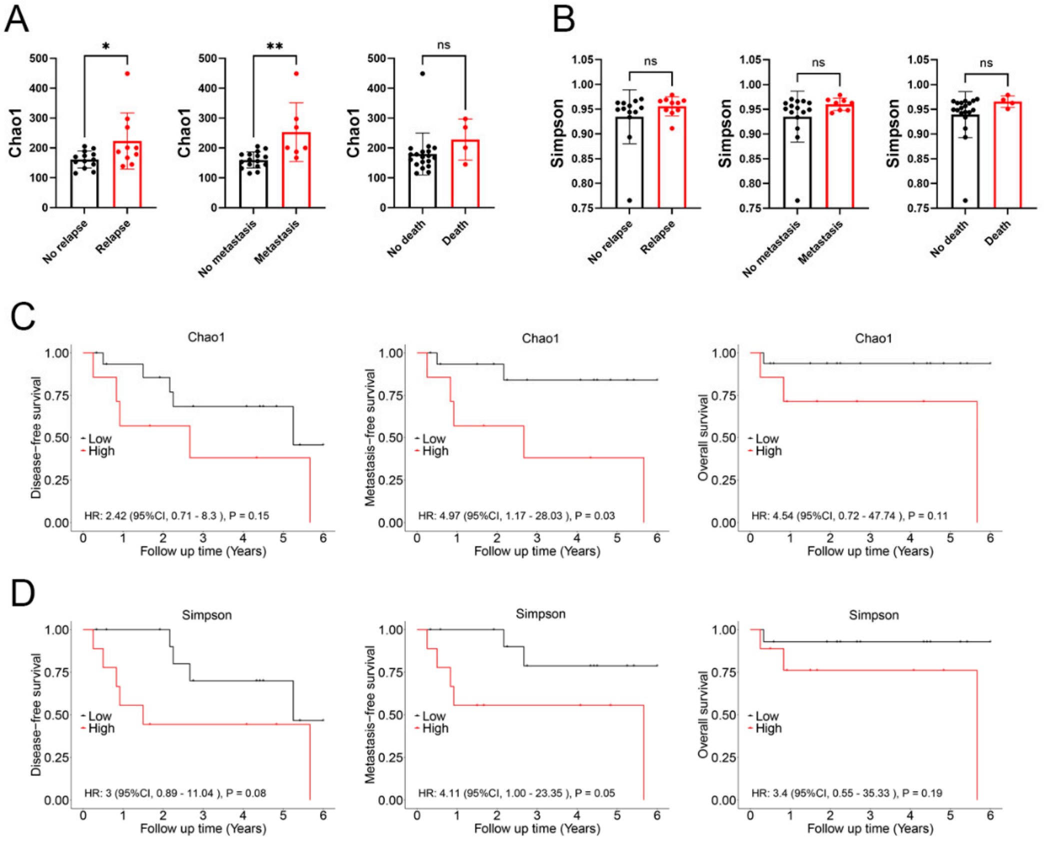

Figure S2. Association of intratumoral microbiota with the prognosis in patients with ACC (N=23). A. The Chao1 index of intratumoral bacteria in ACC patients with or without tumor relapse, metastasis, or death. **B.** The Simpson index of intratumoral bacteria in ACC patients with or without tumor relapse, metastasis, or death. **C.** Kaplan-Meier curves illustrating disease-free survival, metastasis-free survival, and overall survival in ACC patients with a high Chao1 index. P values were analyzed using the log-rank test, and HRs and 95% CIs were calcu-lated using Cox regression analysis adjusted for age, sex, stage and therapy. **D.** Kaplan-Meier curves illustrating disease-free survival, metastasis-free survival, and overall survival in ACC patients with a high Simpson index. P values were analyzed using the log-rank test, and HRs and 95% CIs were calcu-lated using Cox regression analysis adjusted for age, sex, stage and therapy. Data are presented as mean ± SD. $*p < 0.05$, $**p < 0.01$.

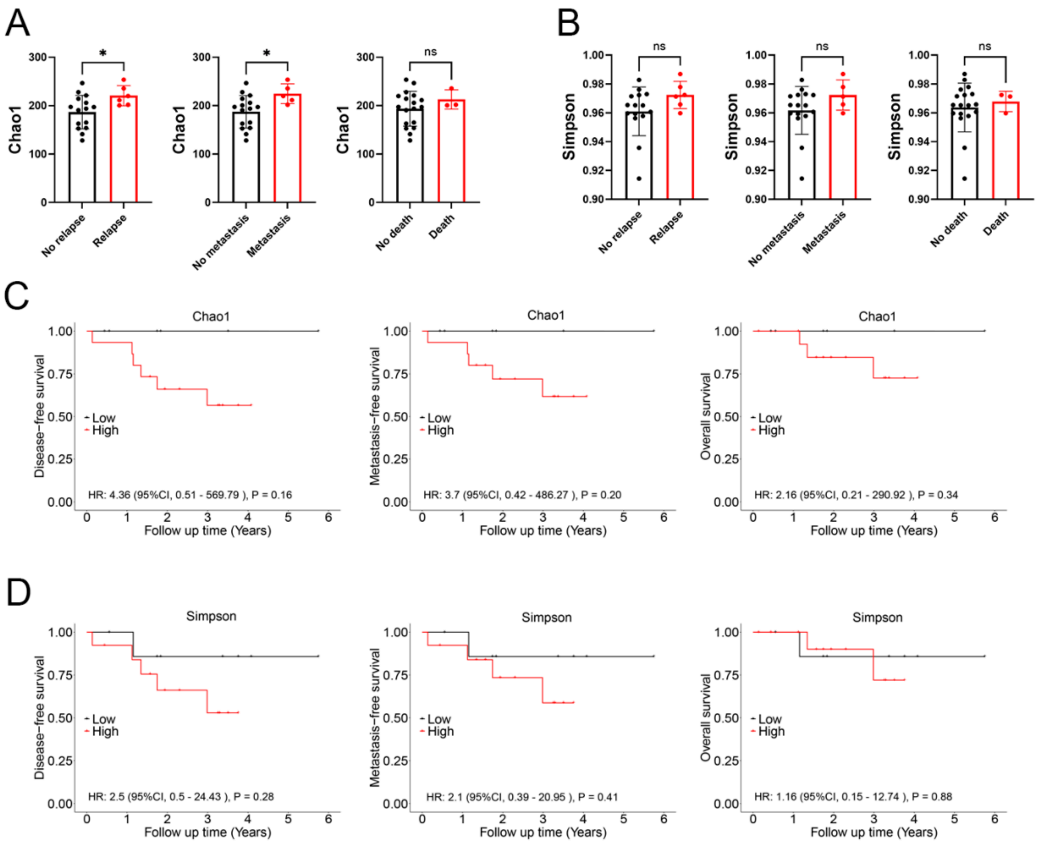

Figure S3. Association of intratumoral microbiota with the prognosis in patients with CXPA (N=21). A. The Chao1 index of intratumoral bacteria in CXPA patients with or without tumor relapse, metastasis, or death. **B.** The Simpson index of intratumoral bacteria in CXPA patients with or without tumor relapse, metastasis, or death. **C.** Kaplan-Meier curves illustrating disease-free survival, metastasis-free survival, and overall survival in CXPA patients with a high Chao1 index. P values were analyzed using the log-rank test, and HRs and 95% CIs were calcu-lated using Cox regression analysis adjusted for age, sex, stage and therapy. **D.** Kaplan-Meier curves illustrating disease-free survival, metastasis-free survival, and overall survival in CXPA patients with a high Simpson index. P values were analyzed using the log-rank test, and HRs and 95% CIs were calcu-lated using Cox regression analysis adjusted for age, sex, stage and therapy. Data are presented as mean ± SD. *p < 0.05, **p < 0.01.

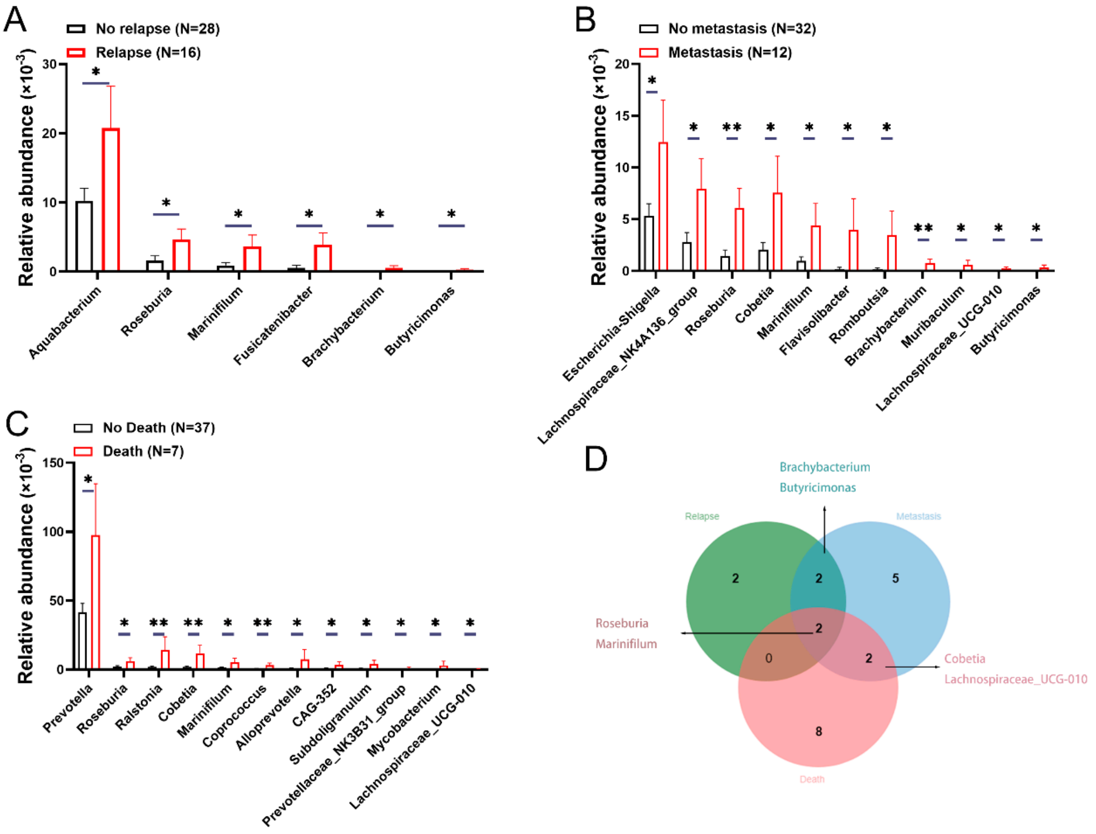

Figure S4. Association of intratumoral microbiota with prognosis in patients with malignant lacrimal tumors (N=44). The altered genera of intratumoral microbiota in patients with or without tumor relapse (**A**), metastasis (**B**), or death (**C**). **D.** Venn diagram comparing the overlapping and distinct intratumoral microbiota across patients experiencing tumor relapse, metastasis, and death. Data are shown as mean ± SD. *p<0.05, **p<0.01.

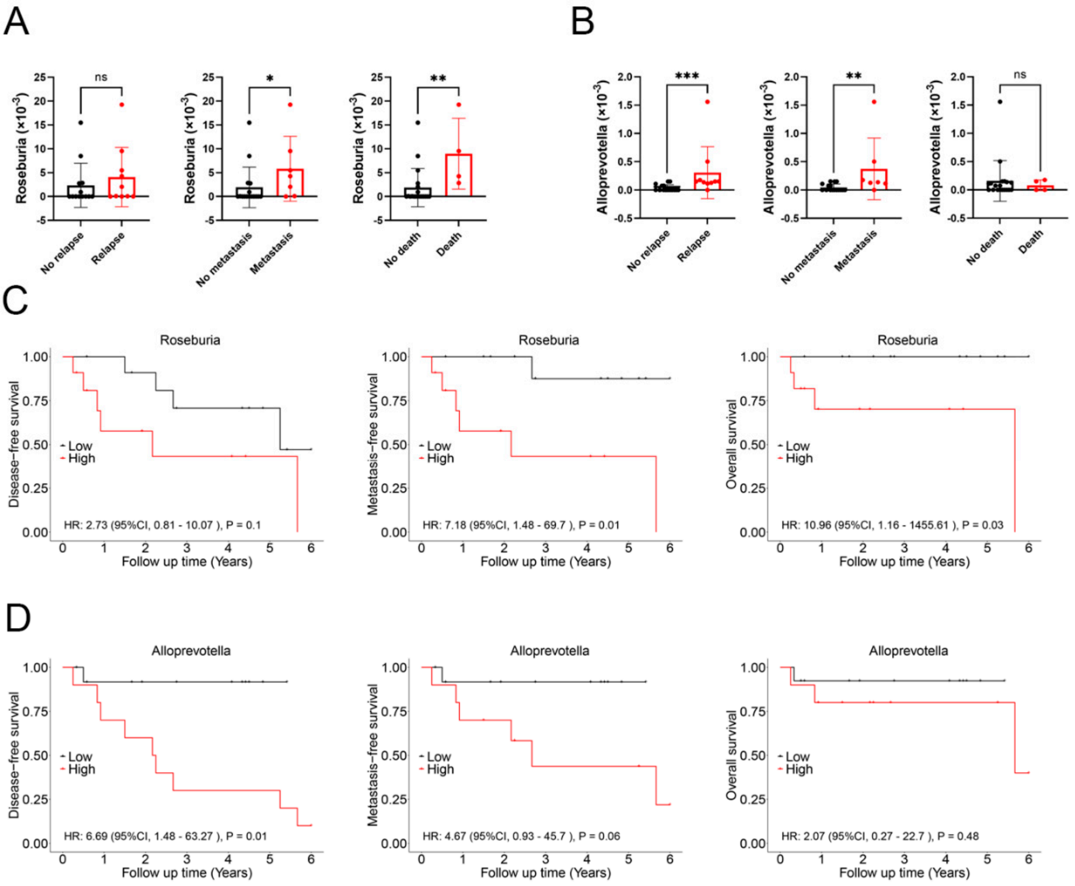

Figure S5. Association of intratumoral microbiota with the prognosis in patients with ACC (N=23). A. The relative abundance of intratumoral *Roseburia* in ACC patients, with or without tumor relapse, metastasis, or death. **B.** The relative abundance of intratumoral *Alloprevotella* in ACC patients with or without tumor relapse, metastasis, or death. **C.** Kaplan-Meier curves illustrating disease-free survival, metastasis-free survival, and overall survival in ACC patients with a high relative abundance of *Roseburia*. P values were analyzed using the log-rank test, and HRs and 95% CIs were calcu-lated using Cox regression analysis adjusted for age, sex, stage and therapy. **D.** Kaplan-Meier curves illustrating disease-free survival, metastasis-free survival, and overall survival in ACC patients with a high relative abundance of *Alloprevotella*. P values were analyzed using the log-rank test, and HRs and 95% CIs were calcu-lated using Cox regression analysis adjusted for age, sex, stage and therapy. Data are presented as mean ± SD. *p < 0.05, **p < 0.01.

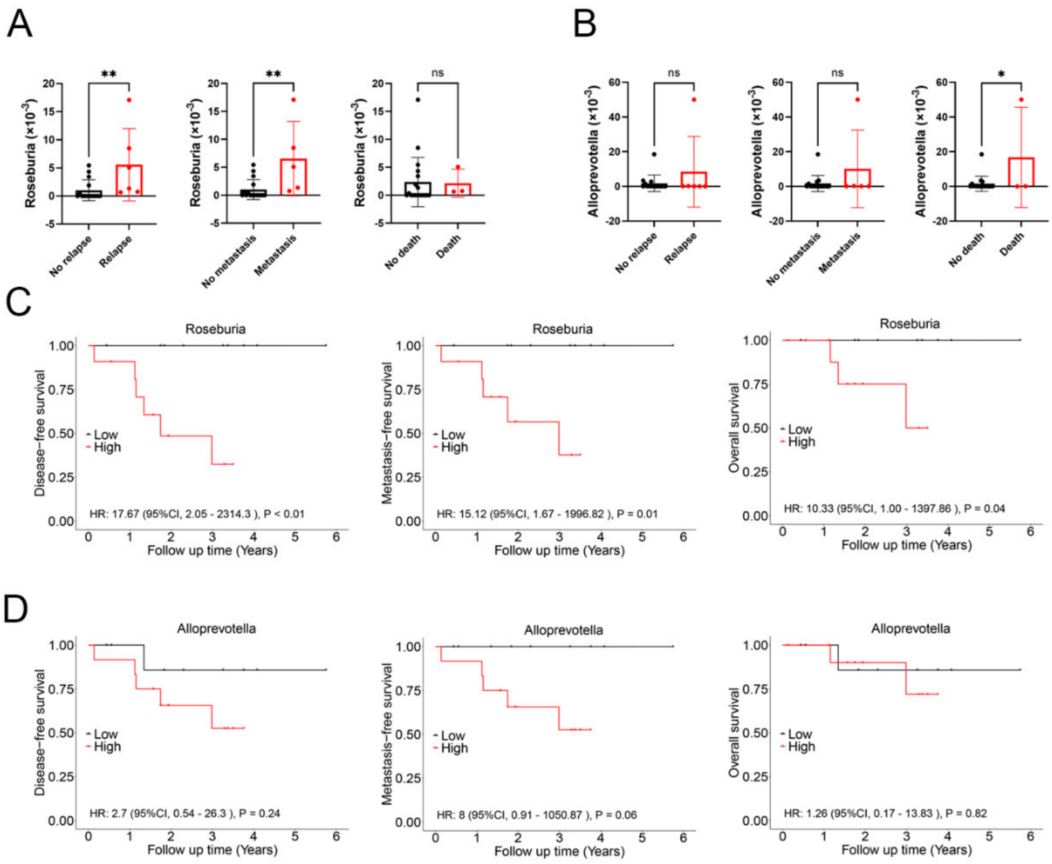

Figure S6. Association of intratumoral microbiota with the prognosis in patients with CXPA (N=21). A. The relative abundance of intratumoral *Roseburia* in CXPA patients, with or without tumor relapse, metastasis, or death. **B.** The relative abundance of intratumoral *Alloprevotella* in CXPA patients with or without tumor relapse, metastasis, or death. **C.** Kaplan-Meier curves illustrating disease-free survival, metastasis-free survival, and overall survival in CXPA patients with a high relative abundance of *Roseburia*. P values were analyzed using the log-rank test, and HRs and 95% CIs were calcu-lated using Cox regression analysis adjusted for age, sex, stage and therapy. **D.** Kaplan-Meier curves illustrating disease-free survival, metastasis-free survival, and overall survival in CXPA patients with a high relative abundance of *Alloprevotella*. P values were analyzed using the log-rank test, and HRs and 95% CIs were calcu-lated using Cox regression analysis adjusted for age, sex, stage and therapy. Data are presented as mean ± SD. $*p < 0.05$, $**p < 0.01$.

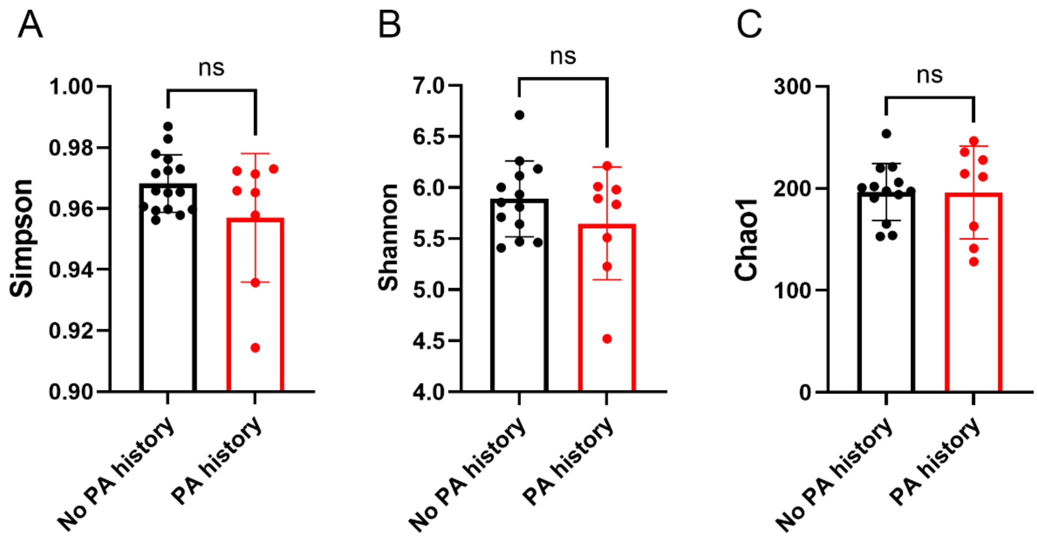

Figure S7. Association of intratumoral microbiota with recurrence and malignancy in PA patients following surgery. A. The Chao1 index of intratumoral bacteria in patients with or without a history of surgery. **B.** The Shannon index of intratumoral bacteria in patients with or without a history of surgery. **C.** The Simpson index of intratumoral bacteria in patients with or without a history of surgery. Data are shown as mean ± SD. ns $p>0.05$.

Table s1. The clinical characteristics of patients with lacrimal gland tumors

**Characteristic**	**ACC(n=23)**	**CXPA(n=21)**	**PA(n=21)**	**IgG4-RD(n=24)**	***P*-value**
**Age**	49.4±12.1	56.6±14.7	47.2±15.9	53.0±19.2	0.31
**Sex**					0.52
Male	12	13	11	17	
Female	11	8	10	7	
**T stage**			NA	NA	0.86
T1-2	18	15	NA	NA	
T3-4	5	6	NA	NA	
**N stage**			NA	NA	0.57
Nx	13	11	NA	NA	
N0	10	9	NA	NA	
N1-3	0	1	NA	NA	
**M stage**			NA	NA	1.0
M0	23	21	NA	NA	
M1-4	0	0	NA	NA	
**Ki67, %**	23.5±19.8	33.3±25.9	NA	NA	0.17
**Chemotherapy**			NA	NA	
Yes	3	0	NA	NA	1.0
No	20	21	NA	NA	
**Radiotherapy**			NA	NA	1.0
Yes	18	15	NA	NA	
No	5	6	NA	NA	

**Table S2. List of significantly different microbiota between ACC, CXPA, PA and IgG4-RD patients.**				
**Genera**	**Groups**	**LDA_value**	**p-value**	**FDR_P**
**k__Bacteria.p__Actinobacteriota.c__Actinobacteria.o__Micrococcales.f__Micrococcaceae.g__Garicola**	**ACC**	**3.706580829**	**0.000729362**	**0.035626512**
k__Bacteria.p__Firmicutes.c__Bacilli.o__Lactobacillales.f__Leuconostocaceae.g__Weissella	ACC	4.198100454	0.008528633	0.216627279
k__Bacteria.p__Proteobacteria.c__Gammaproteobacteria.o__Burkholderiales.f__Comamonadaceae.g__Aquabacterium	ACC	3.616681452	0.022708724	0.343334273
k__Bacteria.p__Actinobacteriota.c__Coriobacteriia.o__Coriobacteriales.f__Atopobiaceae.g__Atopobium	ACC	3.376915272	0.031987044	0.432165375
k__Bacteria.p__Firmicutes.c__Clostridia.o__Oscillospirales.f__Oscillospiraceae.g__UCG_005	ACC	3.019692626	0.033766753	0.446706003
k__Bacteria.p__Bdellovibrionota.c__Bdellovibrionia.o__Bdellovibrionales.f__Bdellovibrionaceae.g__Bdellovibrio	ACC	3.249582878	0.038240449	0.452797304
**k__Bacteria.p__Bacteroidota.c__Bacteroidia.o__Bacteroidales.f__Prevotellaceae.g__Prevotella**	**CXPA**	**4.319553879**	**0.000258848**	**0.032873751**
k__Bacteria.p__Campilobacterota.c__Campylobacteria.o__Campylobacterales.f__Arcobacteraceae.g__Arcobacter	CXPA	3.314572618	0.001568008	0.066378986
k__Bacteria.p__Proteobacteria.c__Gammaproteobacteria.o__Pseudomonadales.f__Moraxellaceae.g__Psychrobacter	CXPA	3.138016703	0.005875651	0.162219073
**k__Bacteria.p__Campilobacterota.c__Campylobacteria.o__Campylobacterales.f__Helicobacteraceae.g__Helicobacter**	**IgG4**	**3.160020711**	**0.000320422**	**0.033697532**
k__Bacteria.p__Firmicutes.c__Clostridia.o__Lachnospirales.f__Lachnospiraceae.g___Ruminococcus__torques_group	IgG4	3.633413687	0.010692191	0.219017454
k__Bacteria.p__Actinobacteriota.c__Actinobacteria.o__Micrococcales.f__Microbacteriaceae.g__Microbacteriaceae	IgG4	3.18160929	0.040002038	0.452797304
k__Bacteria.p__Bacteroidota.c__Bacteroidia.o__Chitinophagales.f__Chitinophagaceae.g__Puia	IgG4	3.746659555	0.040002038	0.452797304
k__Bacteria.p__Verrucomicrobiota.c__Verrucomicrobiae.o__Pedosphaerales.f__Pedosphaeraceae.g__ADurb_Bin063_1	IgG4	3.482330862	0.040002038	0.452797304
**k__Bacteria.p__Bacteroidota.c__Bacteroidia.o__Flavobacteriales.f__Flavobacteriaceae.g__Polaribacter**	**PA**	**3.685240311**	**8.73E-05**	**0.013854371**
k__Bacteria.p__Bacteroidota.c__Bacteroidia.o__Cytophagales.f__Cyclobacteriaceae.g__Marivirga	PA	3.038206988	0.019111617	0.303396918
k__Bacteria.p__Firmicutes.c__Clostridia.o__Peptostreptococcales_Tissierellales.f__Peptostreptococcaceae.g__Filifactor	PA	3.195285269	0.019111617	0.303396918

**Table S3. The relative abundance of significantly different microbiota between patients with or without relapse.**				
**Genera**	**With relapse (n=16)**	**Without relapse (n=28)**	**p-value**	**FDR_P**
**Fusicatenibacter**	0.003935	0.000552	0.016528506	0.614513993982808
**Butyricimonas**	0.000285	8.29E-06	0.035961989	0.614513993982808
**Brachybacterium**	0.000562	5.02E-05	0.037404575	0.614513993982808
**Roseburia**	0.004635	0.001641	0.042920636	0.614513993982808
**Aquabacterium**	0.020762	0.01028	0.047173936	0.614513993982808
**Marinifilum**	0.003635	0.000921	0.050500471	0.614513993982808

**Table S4. The relative abundance of significantly different microbiota between patients with or without metastasis.**				
**Genera**	**With metastasis (n=12)**	**Without metastasis (n=32)**	**p-value**	**FDR_P**
**Roseburia**	0.006121	0.001458	0.002665313	0.52409156811236
**Brachybacterium**	0.000749	4.39E-05	0.00687339	0.52409156811236
**Butyricimonas**	0.000366	1.24E-05	0.012044484	0.52409156811236
**Lachnospiraceae_UCG-010**	0.000246	1.51E-05	0.017878505	0.52409156811236
**Marinifilum**	0.004373	0.000984	0.022674521	0.52409156811236
**Romboutsia**	0.003471	0.000197	0.024879468	0.52409156811236
**Escherichia-Shigella**	0.012472	0.005307	0.026805676	0.52409156811236
**Cobetia**	0.007555	0.002054	0.027542434	0.52409156811236
**Lachnospiraceae_NK4A136_group**	0.007928	0.002796	0.032459289	0.52409156811236
**Flavisolibacter**	0.004008	0.000177	0.040910147	0.52409156811236
**Muribaculum**	0.000606	7.59E-05	0.045353176	0.52409156811236

**Table S5. The relative abundance of significantly different microbiota between patients with or without death.**				
**Genera**	**With death (n=7)**	**Without death (n=37)**	**p-value**	**FDR_P**
**Cobetia**	0.011941	0.001968	0.000639326	0.16302813
**Coprococcus**	0.003264	0.000635	0.00376956	0.312889558125
**Ralstonia**	0.014324	0.001956	0.005145281	0.312889558125
**Prevotella**	0.097664	0.041718	0.011326916	0.312889558125
**Subdoligranulum**	0.004094	0.00061	0.014341064	0.312889558125
**Mycobacterium**	0.003067	2.36E-05	0.017882581	0.312889558125
**CAG-352**	0.003398	0.000857	0.021730266	0.32595399
**Marinifilum**	0.005201	0.001285	0.031093898	0.440496888333333
**Lachnospiraceae_UCG-010**	0.000286	3.86E-05	0.038344314	0.49463931
**Alloprevotella**	0.007499	0.000765	0.03879524	0.49463931
**Roseburia**	0.006052	0.002101	0.042236382	0.512870352857143
**Prevotellaceae_NK3B31_group**	0.001013	0.000207	0.049455463	0.573233775681818

Table S6. The relative abundance of significantly different microbiota between CXPA patients with or without a history of surgery.				
**Genera**	**With PA surgery history (n=8)**	**Without PA surgery history (n=13)**	**p-value**	**FDR_P**
**Acidovorax**	0.018715282	0.007321831	0.02146429	0.268856
**Cobetia**	0.008549206	0.001880688	0.013628567	0.268856
**Arcobacter**	0.000388045	0.007595986	0.036573101	0.268856
**Actinomyces**	0.003349487	1.85883E-05	0.048797257	0.268856
**Dechloromonas**	0.000545732	2.39087E-05	0.039168888	0.268856
**Campylobacter**	0.000700075	0	0.036261987	0.268856
**Butyricimonas**	1.40533E-05	0	0.036879977	0.268856
**Akkermansia**	0	6.16407E-05	0.038887502	0.268856
